# Continuous glucose monitoring in the ICU: clinical considerations and consensus

**DOI:** 10.1186/s13054-017-1784-0

**Published:** 2017-07-31

**Authors:** James S. Krinsley, J. Geoffrey Chase, Jan Gunst, Johan Martensson, Marcus J. Schultz, Fabio S. Taccone, Jan Wernerman, Julien Bohe, Christophe De Block, Thomas Desaive, Pierre Kalfon, Jean-Charles Preiser

**Affiliations:** 1 Division of Critical Care, Department of Medicine, Stamford Hospital, Columbia University College of Physicians and Surgeons, Stamford, CT 06902 USA; 2 Department of Mechanical Engineering, Centre for Bio-Engineering, University of Canterbury, Christchurch, 8140 New Zealand; 3 Clinical Division and Laboratory of Intensive Care Medicine, Department of Cellular and Molecular Medicine, KU Leuven, 3000 Leuven, Belgium; 4 Department of Intensive Care, Austin Hospital, Heidelberg, 3084 VIC Australia; 5 Department of Anesthesia and Intensive Care Medicine, Karolinska University Hospital, Department of Physiology and Pharmacology, Karolinska Institutet, 171 77 Stockholm, Sweden; 6 Academic Medical Center, Meibergdreef 9, 1105 AZ Amsterdam, The Netherlands; 7 Department of Intensive Care, Laboratory of Experimental Intensive Care and Anesthesia (L E I C A), Faculty of Tropical Medicine, Mahidol University, Mahidol-Oxford Research Unit (MORU), Bangkok, Thailand; 8 Department of Intensive Care, Erasme Hospital, Université libre de Bruxelles, 1070 Brussels, Belgium; 9 Karolinska University Hospital Huddinge & Karolinska Institutet, K32 14186, Stockholm, Sweden; 10 Medical Intensive Care Unit, University Hospital of Lyon, Lyon, France; 11 Department of Endocrinology, Diabetology and Metabolism, Antwerp University Hospital, B-2650 Edegem, Belgium; 12 GIGA—In Silico Medicine, Université de Liège, B4000 Liège, Belgium; 13 Service de Réanimation polyvalente, Hôpital Louis Pasteur, CH de Chartres, 28000 Chartres, France

**Keywords:** Glucose, Insulin, Diabetes, Neurointensive care, Monitoring

## Abstract

Glucose management in intensive care unit (ICU) patients has been a matter of debate for almost two decades. Compared to intermittent monitoring systems, continuous glucose monitoring (CGM) can offer benefit in the prevention of severe hyperglycemia and hypoglycemia by enabling insulin infusions to be adjusted more rapidly and potentially more accurately because trends in glucose concentrations can be more readily identified. Increasingly, it is apparent that a single glucose target/range may not be optimal for all patients at all times and, as with many other aspects of critical care patient management, a personalized approach to glucose control may be more appropriate. Here we consider some of the evidence supporting different glucose targets in various groups of patients, focusing on those with and without diabetes and neurological ICU patients. We also discuss some of the reasons why, despite evidence of benefit, CGM devices are still not widely employed in the ICU and propose areas of research needed to help move CGM from the research arena to routine clinical use.

## Background

The story of tight glucose control in the intensive care unit (ICU) dates back to the publication of the first of the Leuven studies [1]. This study showed reduced mortality rates in surgical ICU patients who were managed according to a protocol that strictly controlled blood glucose levels using an insulin infusion. The study was conducted on a background of increasing evidence for a potentially harmful effect of stress hyperglycemia on morbidity and mortality, and triggered a surge of interest among intensivists worldwide who started to pay more attention to blood glucose concentrations. As with other parameters that are regularly monitored in the ICU, the concept of continuous monitoring was soon advanced for blood glucose. It was suggested that this approach would offer benefit compared to intermittent monitoring systems, enabling insulin infusions to be adjusted more rapidly and potentially more accurately because trends in glucose concentrations could be more readily identified. However, despite initial excitement and agreement by most intensivists that glucose concentrations should be kept lower than they used to be (prior to 2001) and that continuous glucose monitoring (CGM) systems may be superior to intermittent measurements for this purpose, only one device is in clinical use in Europe, and none yet in the United States.

In this review, based on dialog among 12 leading experts in this field, we will consider some of the clinical areas of ongoing discussion regarding CGM in the ICU, including the need for different glucose targets in different patient populations, and how this technology can be moved from the research arena into routine clinical use.

## Different targets for different situations

The exciting results of the single-center Leuven studies [1, 2] were not confirmed in subsequent multicenter randomized controlled trials (RCTs) [3–5], even when computerized tools to facilitate tight glucose control were used [6, 7]. However, these multicenter RCTs had several limitations, including that the monitoring technology used may have been associated with analytic inaccuracy; that the monitoring frequency was suboptimal, increasing the likelihood of “missed” hypoglycemic and hyperglycemic events; and that the clinical teams were not as well trained as the Leuven team. These issues led to a variable and often low proportion of time within the targeted blood glucose range. Moreover, RCTs are notoriously difficult to conduct in the heterogeneous ICU population and have rarely demonstrated an impact of any intervention on mortality [8, 9].

Indeed, as the glucose story has unfolded it has become apparent, as in many other areas of intensive care, that one size does not fit all. Different types of patient may have different needs in terms of glucose control, making it difficult to demonstrate overall benefit in large heterogeneous trials. For example, observational data support the importance of glycemic control in patients admitted to the ICU following trauma [10–12]. In the predefined trauma subpopulation of the NICE-SUGAR trial (*n* = 888/6028), there was a trend toward reduced mortality in the patients treated with the “tight” blood glucose target (odds ratio 0.77 (95% confidence interval 0.50–1.18)) [3]. Moreover, patients treated with systemic glucocorticoids (*n* = 1997/6019), an additional subgroup from this trial, also demonstrated a trend toward reduced mortality (odds ratio 0.88 (95% confidence interval 0.66–1.19)) when receiving intensive glucose control [3].

Here we consider two additional groups as examples of categories of patients in whom optimal blood glucose concentrations may differ from those of other types of patient: patients with diabetes and neuro-ICU patients.

### Patients with versus those without diabetes

Few studies have examined the impact of stress hyperglycemia and tight glucose control in critically ill patients with diabetes and there are currently no data from interventional RCTs that have specifically studied this population. Some subgroup analyses from the large multicenter RCTs in general ICU populations reported no differences in effect between patients with and those without diabetes [3, 5, 6], while others demonstrated mortality benefit mainly in patients without diabetes [13]. A post-hoc analysis of the adult Leuven studies revealed no mortality benefit from tight blood glucose control in the subgroup of patients with diabetes, in contrast to all other subgroups, but there was a trend toward reduced morbidity [14].

Observational data suggest that the independent association of hyperglycemia with mortality in the critically ill is robust in patients without diabetes but not so in those with diabetes [15, 16]. Other work has demonstrated that a long time in range (70–140 mg/dl) is independently associated with survival in patients without diabetes but not in those with diabetes [17].

In addition to differences between patients with and without diabetes, there may also be differences depending on the degree of premorbid glycemic control in patients with diabetes. Indeed, the glycemic threshold at which the counterregulatory mechanisms to control blood glucose concentrations are activated is higher in patients with poorly controlled diabetes than in those with well-controlled diabetes or without diabetes [18]. Egi et al. [19] reported that the time-weighted average blood glucose concentration in patients with high glycated hemoglobin (HbA1c) was higher in survivors than in nonsurvivors, suggesting that conventional blood glucose targets may not be appropriate in patients with poorly controlled diabetes. The same group recently reported that the higher the HbA1c level prior to ICU admission, the higher the risk of death in patients with moderate (40–69 mg/dl) and severe (<40 mg/dl) hypoglycemia [20]. Should glucose targets in the ICU therefore be adjusted according to HbA1c levels? Two recent small pilot studies from Australia in critically ill patients with diabetes and HbA1c > 7% tested two glucose targets (6–10 mmol/l (108–180 mg/dl) and 10–14 mmol/l (180–252 mg/dl)) and demonstrated reduced glucose variability [21] and reduced “relative” hypoglycemia (defined as blood glucose values < 30% predicted mean glycemia) [22] in patients with the loose target. A recent single-center, 2-year, before–after investigation evaluated two blood glucose targets: 80–140 mg/dl for patients without diabetes and those with diabetes and HbA1c < 7%, versus 110–160 mg/dl for patients with diabetes and HbA1c > 7%; there was a reduction in severity-adjusted mortality in the patients with diabetes [23].

#### Summary and recommendations/food for thought/future studies

Data therefore suggest that the optimal blood glucose target may be higher in patients with preexisting diabetes compared to those without. However, further study is needed to define the optimal level, because too high a level may also be associated with complications, such as infection. Importantly, most of the published data in this area are from studies using intermittent glucose testing, and newer data using CGM techniques should provide more information including the importance of glucose variability and time in range for patients with and without diabetes.

The effects of insulin resistance also need to be taken into account. HbA1c levels should be measured in all critically ill patients and care taken to avoid relative hypoglycemia in those with poor preadmission glucose control. However, the precise definition of “relative hypoglycemia” and whether it should be based on some measure of chronic baseline glucose or on the acute baseline remain unclear; the time factor over which the relative hypoglycemia occurs may also be important. The achievement of a fixed percentage decrease in blood glucose (i.e., the incidence of “relative hypoglycemia”) will also be influenced by the target glycemic level. A large multicenter trial (NCT02244073) comparing a blood glucose target based on the admission HbA1c value to standard of care has been completed recently.

### Neurointensive care patients

Glucose is particularly important in the brain because the brain has high energy requirements and limited glucose reserves. In microdialysis studies in neuro-ICU patients, tight glucose control was associated with reduced cerebral glucose and an increased risk of cerebral metabolic distress [24–27]. Hypoglycemia can cause secondary brain injury and should be avoided in these patients. However, hyperglycemia can enhance brain injury and various observational studies have demonstrated increased mortality associated with hyperglycemia in patients with traumatic brain injury (TBI) [28–30] and intracerebral hemorrhage [31–33].

Prospective data support the rationale of controlling glucose concentrations within specific ranges in neurological patients. In a subanalysis of 63 patients with isolated brain injury from the first Leuven study [1], van den Berghe et al. [34] reported that insulin therapy targeting a blood glucose of 80–110 mg/dl was associated with reduced intracranial pressure, reduced need for vasopressors, fewer seizures, and better 1-year functional outcomes than a target blood glucose > 200 mg/dl. In a before–after analysis of an ICU database, tight glucose control (targeting blood glucose between 80 and 140 mg/dl) reduced the odds of a poor outcome compared to conventional glucose control (targeting blood glucose < 200 mg/dl) in patients with aneurysmal subarachnoid hemorrhage [35]. However, in a 24-month follow-up of patients with TBI included in the NICE-SUGAR study, neurological outcomes and survival rates were similar in the intensive and conventional groups [36].

Several prospective trials have compared “tight” versus “conventional” glucose control protocols specifically in critically ill patients with neurological conditions [37], including those with TBI [38–41], subarachnoid hemorrhage [42], stroke [43], and neurosurgery [44]. Most of these studies reported increased rates of hypoglycemia in patients in the tight control arm with little or no impact on mortality or neurological outcomes, although some reported reduced infection rates with tight control [40, 42, 44]. These studies are difficult to compare because of different patient populations, and different glucose targets within the tight and the conventional groups. A meta-analysis of 16 RCTs reported that tight glucose control was associated with improved neurological outcomes, but only when the control group target glucose was >200 mg/dl [45].

#### Summary and recommendations/food for thought/future studies

Currently available data therefore suggest that tight compared to conventional glucose control has little impact on neurological or mortality outcomes in neuro-ICU patients, although it may be associated with a decrease in infectious complications. Interestingly, in a retrospective analysis, Meier et al. [46] reported that a blood glucose target of 3.5–6.5 mmol/l (63–117 mg/dl) during the first week in patients with TBI was associated with significantly elevated intracranial pressure, increased norepinephrine requirements, and a trend toward increased mortality compared to a target of 5–8 mmol/l (90–144 mg/dl), whereas in the second week the lower target seemed more beneficial. It may therefore be that glucose concentrations should be kept at higher levels during the early phase of TBI, and possibly other acute neurological conditions, and lower targets used at later stages. Further study is needed to clarify this issue.

Given the possible negative impact of hypoglycemia on secondary brain injury, depending on its severity and duration, the traditional cutoff values for hypoglycemia may need to be reconsidered and increased in these patients. The differences between patients with and without diabetes also need to be taken into account when considering appropriate glucose targets for neuro-ICU patients. There are few data available, but Bosarge et al. [30] reported that the association between hyperglycemia and mortality in patients with TBI was not present in patients with diabetes as defined by known history or admission HbA1c > 6.5%.

## Design of future studies

Quality clinical studies are essential for continued progress in the field of glucose control and different types of study can be considered, the key features and attributes of which are summarized in Table 1.

**Table 1:** Summary of trial designs

Trial design type	Purpose	Limitations	Comments, recommendations
Randomized controlled trial	To determine proof of causality	Heterogeneous patient populations reduce ability of the trial to ascertain differences in treatment effect. Logistic burden and cost	Participating centers should have a phase-in period that allows demonstration of the capacity of the center to perform the study safely and effectively. Use of a smaller, well-defined, and homogeneous population may increase the probability of determining a true treatment effect
Observational study	Hypothesis generation	Do not provide proof of causality	Collaborations among centers with large databases. Standardization of reporting metrics
Individual patient meta-analysis	To avoid the limitations and complications inherent in comparing disparate studies. Allows interpretation of patient-specific events that would not normally be identified in aggregate trial data	Hugely time and resource intensive	The analysis will be confounded by the same limitations as present in the original trials
Cluster randomization	To reduce variations in process of care for complex interventions as a confounding factor in randomized controlled trials	Requires more complex statistical methodology to account for the effects of clustering	May be particularly suitable for complex interventions, such as glucose control in the critically ill

### Randomized controlled trials

The apparent failure of multicenter RCTs to reproduce the positive findings of the original Leuven studies has dampened enthusiasm for tight glucose control. However, although RCTs remain the best way to take into account the multiple factors that can influence the response to any therapeutic intervention, this study design has known limitations in the heterogeneous critically ill patient population [9]. Moreover, glycemic control is different in many ways from other interventions in the ICU, including that it is highly dependent on the chosen protocol and the training (and enthusiasm) of the staff operating the protocol. Glycemic control is also a continuous intervention, not a one-off event, such as, for example, aspirin administration in myocardial infarction. Therefore, its quality throughout the process of care is crucial, as well as more difficult to obtain. Because of the complexity of managing blood glucose during the course of critical illness, we propose that study design should include a phase I period in which the study centers adopt the intervention and show they are capable of achieving an adequate time in the targeted blood glucose range. Only after this ability has been demonstrated should a center be allowed to participate in randomization. Regular review of glucose control performance throughout the trial is also required.

### Observational studies

Observational studies remain a key method to generate hypotheses for future clinical trials. As such, recent studies have begun to explore more personalized approaches to glycemic control based on observational data. For example, in a recent, prospective before–after study, Krinsley et al. [23] studied different blood glucose targets in patients with and without diabetes and in patients with diabetes and HbA1c levels <7% and >7%, based on results from observational studies [16, 19, 47]. They compared a blood glucose target of 90–120 mg/dl for all patients in the “before” period with a dual target in the “after” period according to the presence of poorly controlled diabetes (80–140 mg/dl for patients without diabetes and patients with diabetes and HbA1c < 7%, and 110–160 mg/dl for patients with diabetes and HbA1c > 7%). The results showed similar mortality rates in the two periods for nondiabetic patients, but decreased severity-adjusted mortality in the “after” period in both groups of diabetic patients.

Large databases could also be used to explore questions related to glycemic control, and collaboration between centers should be encouraged to stimulate further research in this field. Ideally such databases should be large, and contain essential demographic data, severity scores, hospital outcomes, a complete set of glucose measures including the key glucose metrics, nutritional information, and HbA1c concentrations. As experts in this field reach agreement on the optimal metrics and outcomes that should be reported [48, 49], studies will begin to include a standard set of data, making it easier to combine datasets and create large databases for analysis.

### Individual patient data meta-analysis

Meta-analysis is a statistical technique in which data from multiple trials are combined. However, when the trials included in a meta-analysis include heterogeneous groups of patients, meta-analysis may be impossible to conduct or give inaccurate results. An alternative approach is to perform individual patient data meta-analysis [50]. This technique has the advantages that greater power is provided than by aggregate study meta-analyses, primary data can be validated, and certain analyses which can only be conducted on individual data (e.g., time-to-event analyses) can be performed. However, such studies involve a huge amount of work synthesizing the individual raw patient data and returning to the original data if certain variables are not recorded in the publication. An individual data meta-analysis of RCTs in this field has recently been completed and the results are eagerly awaited. Nevertheless, this analysis will be confounded by the same limitations as the original RCTs, including, but not limited to, a short time in the targeted blood glucose range and high rates of hypoglycemia among treated patients.

### Cluster randomization

Strict glucose control is complex and involves several consecutive steps, each of which contains possible sources of variability, from the technique used to measure the blood glucose to the algorithm used to adjust the insulin dose, through to the training and motivation of the staff operating the system [51]. As such, cluster-randomized trials, in which groups of individuals rather than single individuals are randomized to an intervention, could represent an advantage over individual randomized trials, reducing the impact of differences in technique among different units. The disadvantages of this approach are that more complex statistical methodology is needed to account for the effects of clustering and more individuals may be needed overall to achieve the same statistical power as in an individual RCT. In a cluster-randomized study in a department of intensive care comprised of four units, patients in two of the units were randomized to standard glucose management and the patients in the two other units were randomized to CGM-guided glucose control. The randomized units’ treatment allocation was crossed over every month. The results showed similar glucose control in terms of time in range, but the time spent with glucose values < 70 mg/dl was reduced in the CGM-guided patients [52].

## Moving forward … and how to keep the industry on board?

There is now a broad base of evidence to support the use of CGM devices as a means of facilitating glucose control and decreasing nursing workload in ICU patients. Yet, despite more than $700 million spent by the industry on developing CGM devices, they remain largely experimental in the ICU and although several have FDA approval for use in noncritically ill patients, the only device that received FDA approval for critically ill patients is not marketed and is not in clinical use. Only one device is currently in clinical use in Europe [53].

So what are the priorities for the future in terms of encouraging more widespread use of these devices and ensuring continued industry investment such that FDA/CE approval can be obtained?

1. Accuracy is clearly important (and essential in terms of licensing). Many of the devices reported in peer-reviewed literature (using different probe location sites and measurement techniques) fail to meet the mean absolute relative difference (MARD) point accuracy standard adopted by a previous consensus panel [54]. One exception is the Optiscanner® (OptiScan Biomedical Corporation, Hayward, CA, USA), which has been reported to have MARD < 10% [53, 55], although other consensus requirements for CGM devices were not met or not reported. Importantly, comparisons, including MARD and Clark error grids, are based on evaluation of differences in values at a single time point, and do not take the trend factor into account. Yet the trends in glucose concentrations provided by CGM can influence insulin dosing and thus glycemic control. Thus, when comparing CGM devices, trend accuracy as well as point accuracy needs to be compared and reported. These assessments should be independent because one can have perfect trend correlation and poor point accuracy if the values are simply shifted. One possible method of reporting trend accuracy independently of point accuracy is the trend compass [56].
  When considering accuracy in CGM, the degree of sensor drift should also be quantified and taken into account. Drift is the tendency of such devices to report increasingly erroneous values due to changes in sensor condition or patient milieu at the insertion site [57, 58] and may mask clinically important trends in glucose concentrations.
2. As each new study is published, we learn more about how current systems can be improved. For example, a recent study reporting on the safety, accuracy, and reliability of an intravenous blood glucose system demonstrated that more than half of the sensors inserted had to be removed before the end of the planned 72-hour study period [59]. Similarly in a RCT comparing the use of subcutaneous CGM with frequent point-of-care measurements, only 177 of the 1954 patients initially screened were finally included [60]. Also, in a study of an intravenous microdialysis CGM technique, the authors were surprised at the number of patients who did not have suitable peripheral veins to use for insertion [61]. These practical issues are important and need to be reported routinely so that they can be resolved and the devices improved. Devices (monitoring system and algorithms) also need to be user-friendly for the nurses who will be operating them, not just for research staff.
3. The evidence base supporting the clinical effectiveness and efficiency of these systems (CGM plus algorithm) in ICU patients is still small and a pragmatic evaluation of systems in the clinical setting may facilitate their more widespread adoption. In-silico simulation has demonstrated that increased frequency of monitoring improves blood glucose control as assessed by reduced occurrence of hypoglycemia, hyperglycemia, and glucose variability [62, 63]. Importantly, these types of analyses assess not only the device but the whole process, including the protocol used to adjust insulin infusion and the nurses operating it, key considerations when thinking about introducing such a system into your ICU.
4. Cost is a key concern in the introduction of any new technology and there are few cost-effective data for CGM systems [60]. A potential method of reducing the costs associated with CGM is to combine other parameters that require accurate monitoring (e.g., blood lactate and hemoglobin) within the same system. An alternative is to encourage collaborative research among companies producing various components of the glycemic control process (e.g., CGM device, software algorithm, etc.). Ideally, to reduce costs involved when changing the insulin algorithm, devices should not be restricted for use with a single algorithm.
5. Further study is needed to define optimal glycemic targets (ranges) in general ICU populations and in some selected populations (e.g., patients with diabetes, neurological injury, trauma, or sepsis), and to consider at what time point in the disease process these targets are most important. In addition, the influence of the feeding regimen on the effectiveness of strict glucose control remains unclear. The use of GCM in such studies will help provide answers to these questions.

## Conclusion

We believe that the prevention of severe hyperglycemia and hypoglycemia should be considered a strong objective in all settings involving critically ill patients and that CGM can contribute to safe, effective glucose management. CGM, when combined with a validated insulin infusion protocol that minimizes glycemic variability, can help improve patient outcomes and reduce workload, which may be cost-effective. Once reliable devices with acceptable point accuracy are available at reasonable running costs, CGM will be useful in all critically ill patients receiving strict glucose control. The optimal blood glucose target remains unclear and may depend on the studied patient population. As with any monitoring device, CGM cannot per se improve outcomes, but must be combined with an effective algorithm and trained, dedicated staff.

## Abbreviations

CGM: Continuous glucose monitoring
HbA1c: Glycated hemoglobin
ICU: Intensive care unit
MARD: Mean absolute relative difference
RCT: Randomized controlled trial
